# Identification of Breast Cancer LCK Proto-Oncogene as a Master Regulator of TNBC Neutrophil Enrichment and Polarization

**DOI:** 10.3390/ijms241713269

**Published:** 2023-08-26

**Authors:** Fatma Al Qutami, Walaa Al Halabi, Mahmood Y. Hachim

**Affiliations:** Department of Medicine, Mohammed Bin Rashid University of Medicine and Health Sciences, Dubai P.O. Box 505055, United Arab Emirates; fatma.alqutami@mbru.ac.ae (F.A.Q.);

**Keywords:** in-silico, TNBC, neutrophils, breast cancer, immune deconvolution

## Abstract

The role of neutrophils in breast cancer shows that the N1 proinflammatory subtype can suppress and attack the tumor. In contrast, the N2 pro-tumor subtype aids the tumor in its survival, progression, and metastasis. Recently, more focus has been directed to the role of innate myeloid cells, specifically neutrophils, in regulating the responses of lymphoid populations both in the progression of cancer and in response to therapy. However, the exact crosstalk between breast cancer cells and neutrophils is poorly understood. In this work, we used in-silico assays to investigate the role of the bidirectional effect of neutrophils on metastatic TNBC. Our reanalysis of publicly available data reveals that most TNBC’s classified within the CE2 subtype are leukocyte-poor and have four major cell types in their ecotypes: dendritic cells, macrophages, fibroblasts, and epithelial cells. Further immune deconvolution of these patients revealed that a few cells significantly differed between groups, including macrophages, neutrophils, and T cells. All BC showed lower infiltrating neutrophils compared to healthy surrounding tissue. Treated TNBCs improved the count of infiltrating neutrophils in TNBC. Most TNBC patients have a unique CE2 ecotype, characterized by more basal-like epithelial cells, more neutrophils, and fewer mononuclear lymphocytes (B cells, macrophages M1, T cell CD4+ (non-regulatory), and T cell CD8+ and T regs). This can be related to our finding that CE2 TNBCs are characterized by a lower LCK and higher ERBB2, and their top DEGs are related to leukocyte activation and NFKB pathway.

## 1. Introduction

Breast cancer (BC) is predominant in women, specifically postmenopausal women. The incidence rates have increased in the past several years, affecting 2.26 million females in 2020 and 2.5 million new cases predicted by 2025 [1,2,3,4]. BC is expected to represent 31% of all new cancer incidences and account for 15% of deaths in 2022 [5]. Data from the Global Cancer Observatory (GCO) reveal that most incidences occurred in Asia, with a younger incidence rate (avg. age 30 years) compared to Europe and North America (41–60 years of age), coinciding with the pre- and postmenopausal period [2,3]. BC is the most common cancer in the United Arab Emirates (UAE) [6] and the second leading cause of death among women due to the advanced stage of presentation with metastasis [7]. Remarkably, 41.9% of breast cancer cases in the UAE were diagnosed before the age of 49 years [8], and 26.9% had triple-negative BC (TNBC) [6]. Due to its high levels of tumor mutational burden, TNBC is an immunogenic subtype of breast cancer with specific immune cell infiltrates which might benefit from immunotherapy [9]. In TNBC, combining cancer immunotherapy with chemotherapy was effective in advanced and early setting phase 3 clinical trials [10]. Nevertheless, increased infiltration of neutrophils in tumors was associated with poor prognosis and limited efficacy of such immune therapeutics [11]. Further understanding of the exact role of neutrophils in this aggressive cancer development and response is warranted.

Recent studies have shown that neutrophils play a role in tumor progression, metastasis, and survival, making them a potential therapeutic target [12]. Neutrophils are polymorphonuclear leukocytes of myeloid origins and are the first cells recruited at the site of inflammation or damage [13]. Neutrophils can eliminate pathogens and modulate inflammation by using different mechanisms, such as phagocytosis, deposition of neutrophil extracellular traps, and secretion of antibacterial proteins [14,15]. Neutrophils infiltrating tumors are called tumor-associated neutrophils (TANs). Furthermore, similar to macrophages, TANs can be classified into the N1 proinflammatory and the N2 anti-inflammatory subtypes, or high-density neutrophils and low-density neutrophils, based on functional characteristics [16]. N1 TANs are more antitumor in function, while N2 TANs favor tumor growth, angiogenesis, and metastasis [17]. Recently, more focus has been directed to the role of innate myeloid cells, specifically neutrophils, in regulating the responses of lymphoid populations both in the progression of cancer and in response to therapy [18]. However, the exact crosstalk between breast cancer cells and neutrophils is poorly understood. In this work, we used in silico assays to investigate the role of the bidirectional effect of neutrophils on metastatic TNBC.

## 2. Results

### 2.1. Leukocyte-Rich BC Showed a Poor Prognosis

Having a comprehensive analysis of the TME is the first step to delineating the exact correlation between tumor cell-intrinsic differences in terms of their expression pattern and the differential presence of immune cells in different molecular subtypes of breast cancers [18] and how each ecotype can affect the overall survival and cancer progression. We used an Ecotyper, a framework for systematically identifying cell states and cellular communities (ecotypes) from bulk, single-cell, and spatially resolved gene expression data using TCGA [19]. 

**Figure 1:**
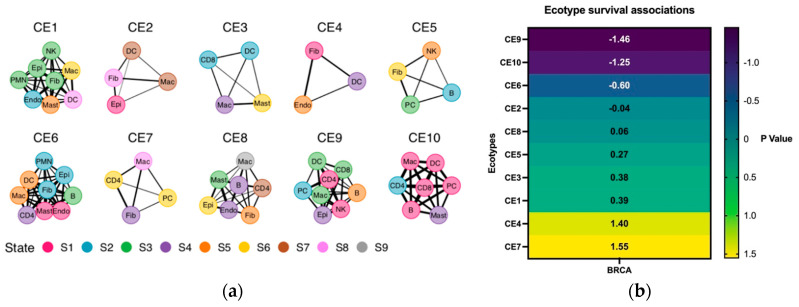
Ecotypes of breast cancer showed (**a**) the cell populations involved in each ecotype and (**b**) their different survival associations of each ecotype in breast cancer.

Our analysis showed that BC survival depends on the ecotype rather than the molecular subtype. For example, patients with BC that showed CE9 and CE10 ecotypes have a significant negative association with survival. On the other hand, CE7 and CE4 showed a positive correlation with survival (Figure 1b). CE9- and CE10-high tumors are proinflammatory (i.e., leukocyte-rich) and characterized by higher immunoreactivity [20]. This goes with a previous note that breast cancer with an increased immune response is associated with aggressive cancer biology [21] and shows the importance of the role of immune cells in determining overall cancer development and outcomes.

### 2.2. The Majority of TNBCs Have a Low Number of Leukocytes

The next question was whether or not the molecular subtypes showed a homogeneous infiltration of immune cells and TME regarding ecotype. Using bulk RNA-seq data, the TCGA-BRCA samples were classified into different ecotypes (Table 1). Interestingly, most TNBCs (75/111, 67.6%) fell into the CE2 ecotype. Likewise, the CE2 ecotype mainly comprised TNBCs (75/87, 89.7%). 

Unlike CE9- and CD10-high tumors, the CE2-high tumors had few leukocytes, indicating the lack of APCs and other cells recruited during inflammation, such as neutrophils. Instead, CE2-high tumors were rich in basal-like epithelial cells but were lymphocyte-deficient and strongly linked to a higher risk of death [19]. 

### 2.3. Treated TNBC Showed Some Increase in Total Tumor Immune Cells

**Figure 2:**
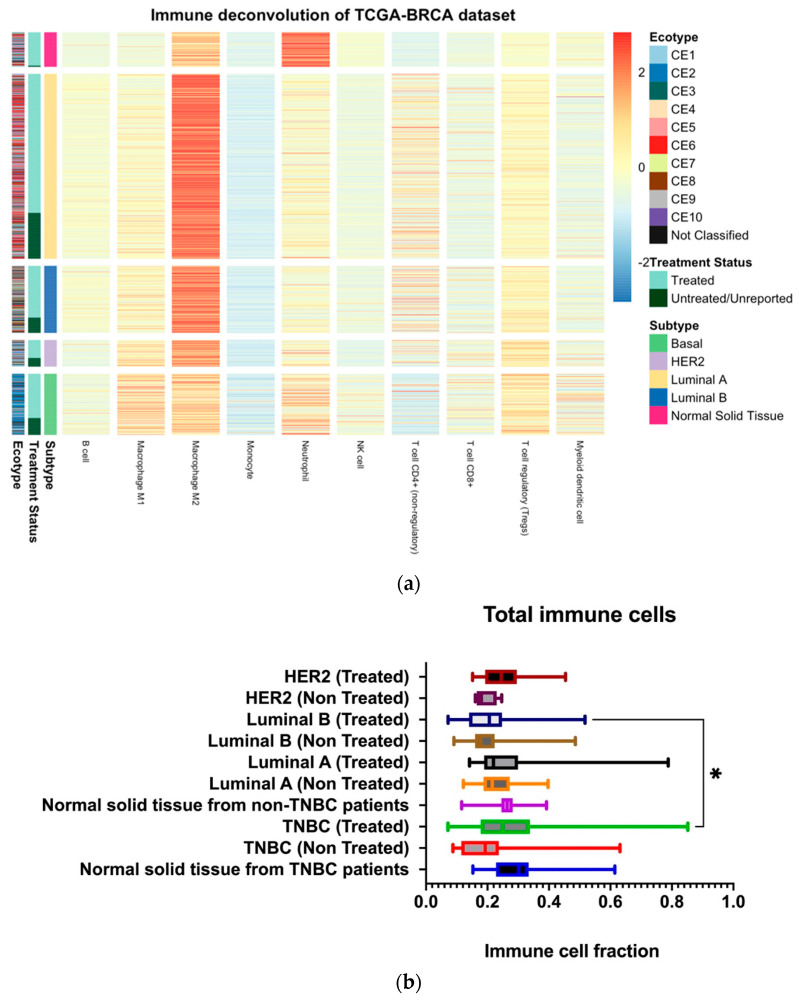
(**a**) Immune cell deconvolution of bulk RNA-seq data of TCGA breast cancer patients based on subtypes. (**b**) Statistical analysis of all immune cell fractions showed that luminal B-treated and TNBC-treated patients had different amounts of immune cell fractions. The asterisk (*) shows statistical significance of *p* = 0.05.

Immune cell deconvolution was carried out on each breast cancer subtype, and groups were also classified based on the treatment received. Then, the mean fractions of each subtype were plotted (Figure 2a) and analyzed statistically. Regarding overall immune cell types, fractions were almost equal in all molecular subtypes, and there was no effect of therapy on the total immune cells in cancer, except for treated TNBC and luminal B patients (Figure 2b). However, treated TNBC showed higher immune cells than treated luminal B, which might indicate that manipulating the local immune cells can be part of the overall effect of therapy on TNBC. 

### 2.4. CE2 TNBC Has Specific Immune Cells Preference with Higher Infiltrating Neutrophils

**Figure 3:**
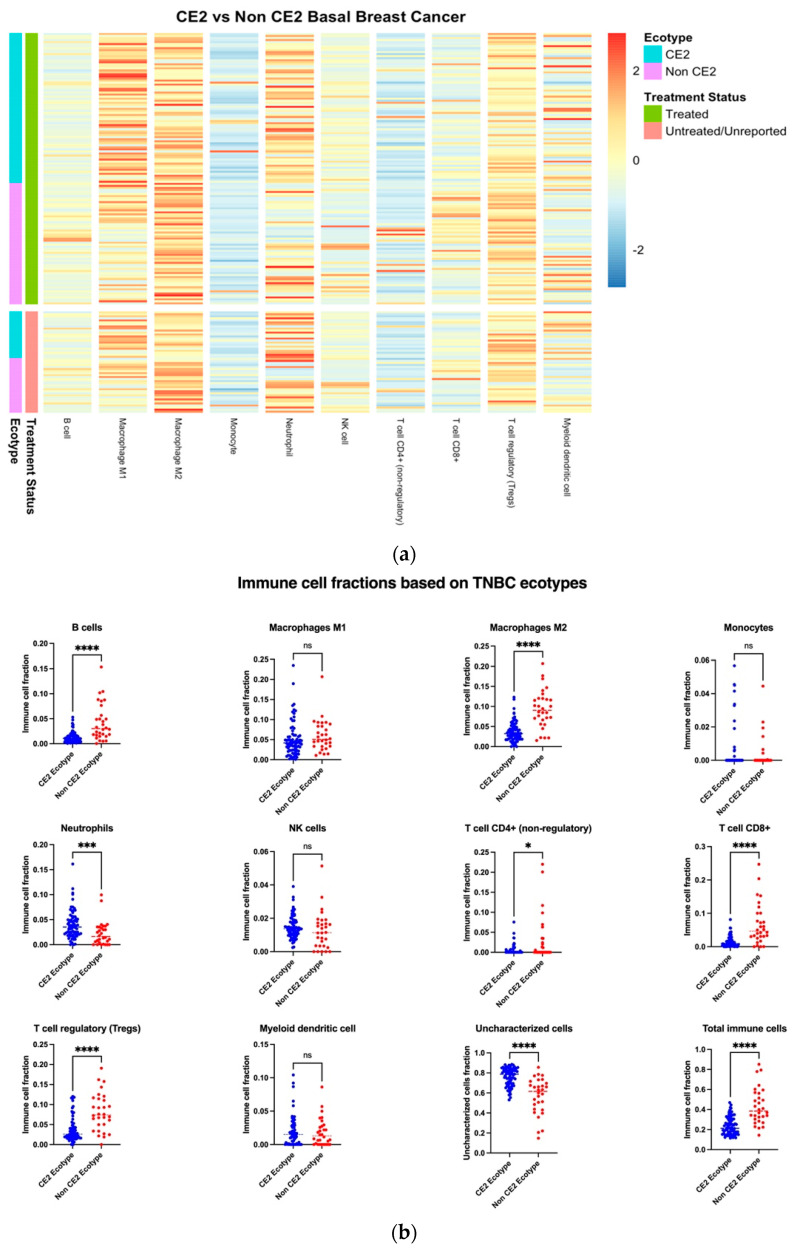
Immune cell deconvolution based on (**a**) ecotype showed higher immune cell fractions in non-CE2 ecotypes. (**b**) Further statistical analysis using the Mann–Whitney test showed that only six immune cell types significantly differed between CE2 and non-CE2 ecotypes. Asterisk shows statistical significance as follows: * means *p* ≤ 0.05; *** means *p* ≤ 0.001; **** means *p* ≤ 0.0001; and ns means not significant.

As shown in Figure 1, CE2 breast cancer patients showed a negative correlation with survival, so we focused later on CE2 TNBC versus non-CE2 TNBC to understand this molecular subtype. TNBC samples were grouped as CE2 ecotype (*n* = 75) and non-CE2 ecotype (*n* = 36) based on EcoTyper classification, and their immune cell scores were compared. There was a higher fraction of immune cells in the non-CE2 ecotype compared to the CE2 ecotype (Figure 3a,b). However, the Mann–Whitney test revealed only six cell types: B cells, macrophages M1, T cell CD4+ (non-regulatory), T cell CD8+, and T regs were statistically lower in CE2 compared to non-CE2 TNBC (Figure 3b). Interestingly, only neutrophils were significantly higher in CE2 than non-CE2. Both tumor-infiltrating neutrophils (TINs) and lymphocytes (TILs) represent the inflammatory cells in the tumor microenvironment where TINs are usually considered typically as pro-tumor while lymphocytes are found to be associated with a better clinical response in carcinomas [22]. Furthermore, the higher fractions of uncharacterized cells in the CE2 ecotype indicate a highly heterogeneous population and might be from the enriched basal-like epithelial cells in the TME of TNBCs. 

**Figure 4:**
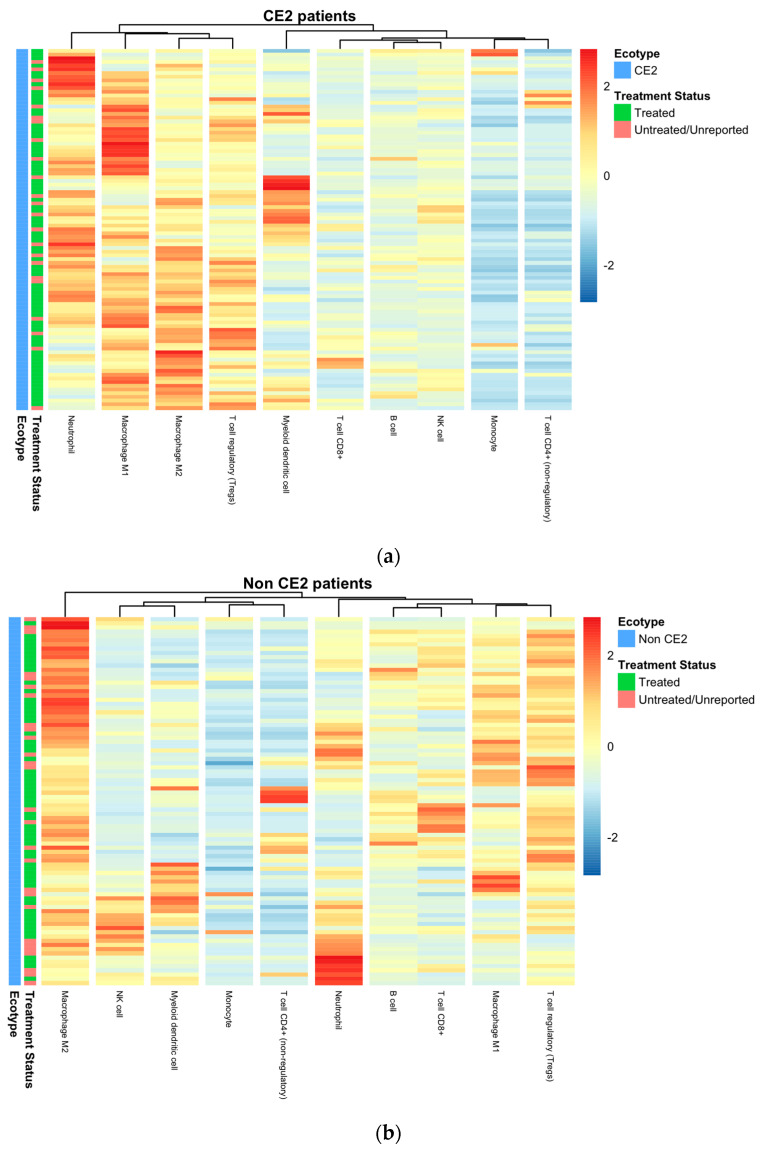
Hierarchical clustering of immune deconvoluted fractions of (**a**) CE2 showed clustering of neutrophils with macrophages and T regs, while (**b**) non-CE2 group clustering exhibited a different pattern with neutrophils clustering close to B cells.

Hierarchal clustering of deconvoluted immune fractions in CE2 TNBC patients showed close and tight clustering of neutrophils with macrophages (M1 and M2) and T regulatory cells, as shown in Figure 4. This was expected as most of these cells are immunosuppressive and can act along with myeloid-derived suppressive cells and tumor-associated dendritic cells in immune resistance and evasion [23]. On the other hand, non-CE2 TNBC patients had clusters of neutrophils with B cells, M1 macrophages, and T cells (CD8+ and Tregs). This clustering is due to neutrophils acting as effector cells, similarly to APCs, in order to promote B cells in initiating T cell responses [24]. 

### 2.5. Neutrophils Are Reduced in Tumor Tissue Compared to Adjacent Normal Tissue

**Figure 5:**
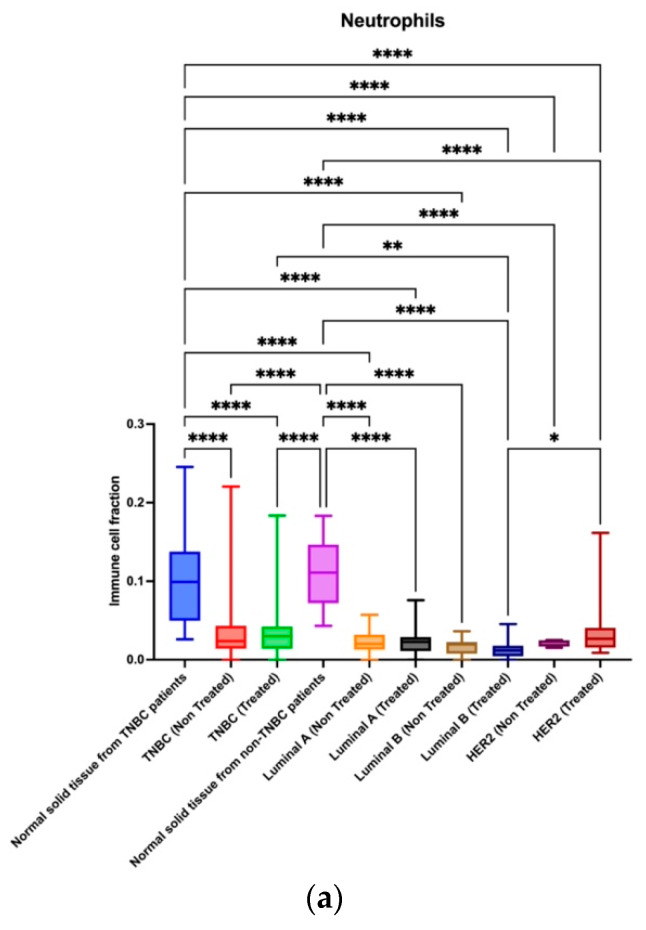

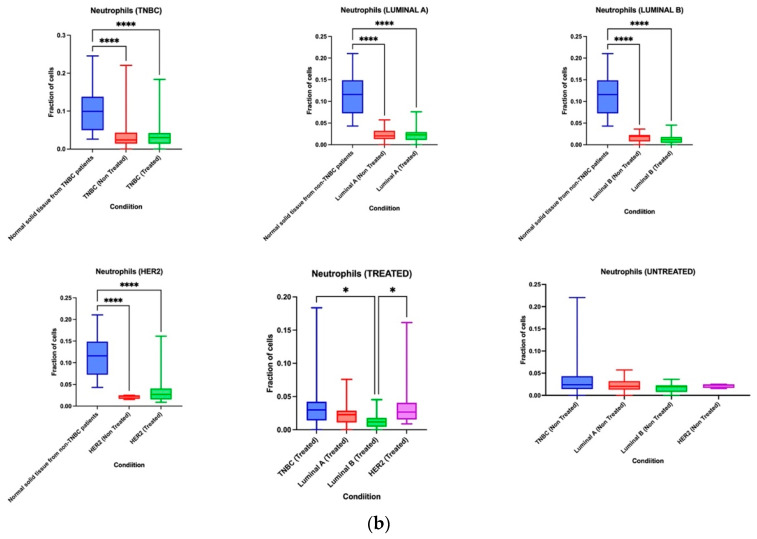
(**a**) The five immune cell types were found at different levels in breast cancer subtypes and depending on whether treatment was received or not. (**b**) The neutrophil expression between the different breast cancer subtypes showed that neutrophils were higher in non-tumor cells than tumor cells. Asterisk shows statistical significance as follows: * means *p* ≤ 0.05; ** means *p* ≤ 0.01; **** means *p* ≤ 0.0001; and ns means not significant.

However, individual cell types such as macrophages M1 and M2, neutrophils, T cell CD4+ non-regulatory, and T cells regulatory were found to have different infiltrations in different BC groups (Figure 5a). Neutrophils appeared to be reduced in tumor tissue compared to normal solid tissue, regardless of the subtype (Figure 5b). This lack of significance in immune cell fractions is due to the varying fractions of immune cells in patients within the same group and is the basis of the different ecotypes within a single tumor subtype. 

### 2.6. CE2 TNBC DEG Are Enriched with NFKB Pathways

**Figure 6:**
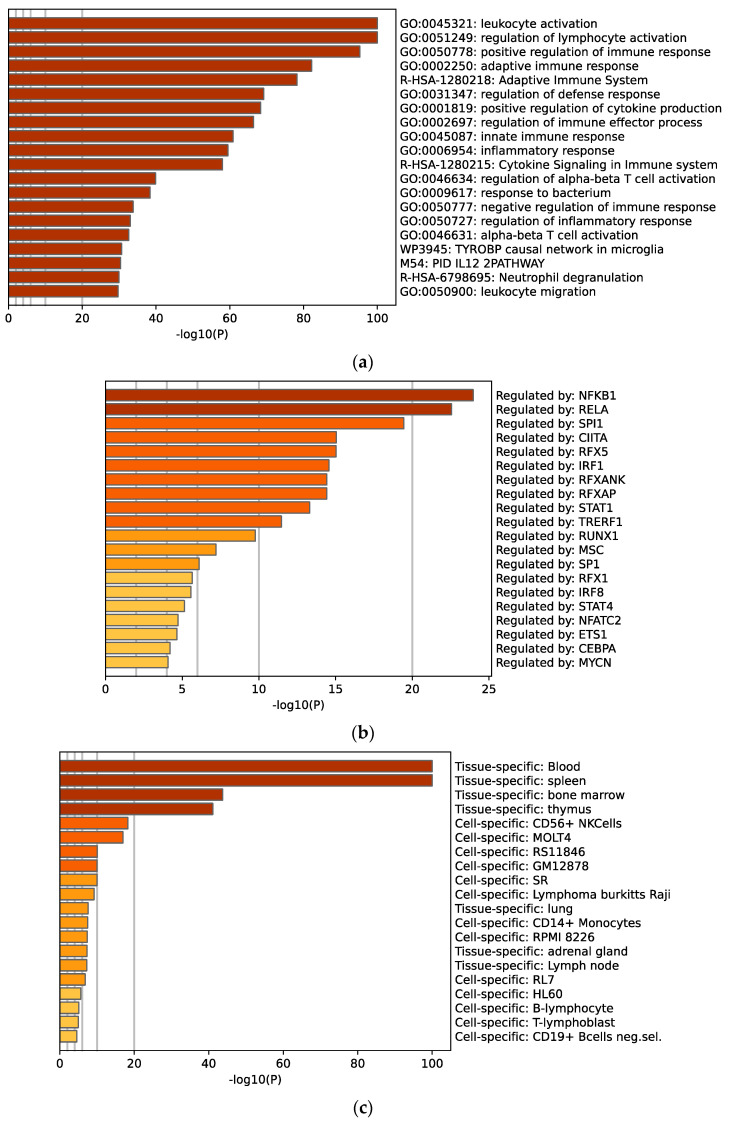
Metascape analysis of the mRNA that is significant in CE2 and non-CE2 ecotypes of TNBC patients in cBioPortal using the TCGA database. (**a**) Pathway enrichment analysis of these differentially expressed genes, alongside (**b**) their transcription factor-target regulatory interactions, as well as (**c**) the Pattern Gene Database.

Pathway enrichment of the differentially expressed gene between the ecotypes using the Metascape database reveals different immune pathways’ involvement (Figure 6a). Major pathways include leukocyte activation and regulation of different immune responses. Furthermore, data show that most of these genes are regulated by NFKB1 and RELA (Figure 6b). Studies have shown that NFKB1 has a role in tumor progression, metastasis, and resistance to chemotherapy in breast cancer patients [25,26]. Furthermore, RELA, a subunit of the NFKB1, has also been identified as a target for preventing NFKB1-driven tumors, as this subunit is more highly expressed in breast cancer compared to adjacent tissues [26,27]. The Pattern Gene Database (PaGenBase) shows that most of these genes are specific to the blood, spleen, and bone marrow (Figure 6c). This is because the major difference between the two ecotypes is the immune cell composition generated from the bone marrow and spleen and circulating in the blood.

### 2.7. CE2 TNBC Has a Specific Genomic, Transcriptomic, Proteomic, and Microbiome Signature

**Figure 7:**
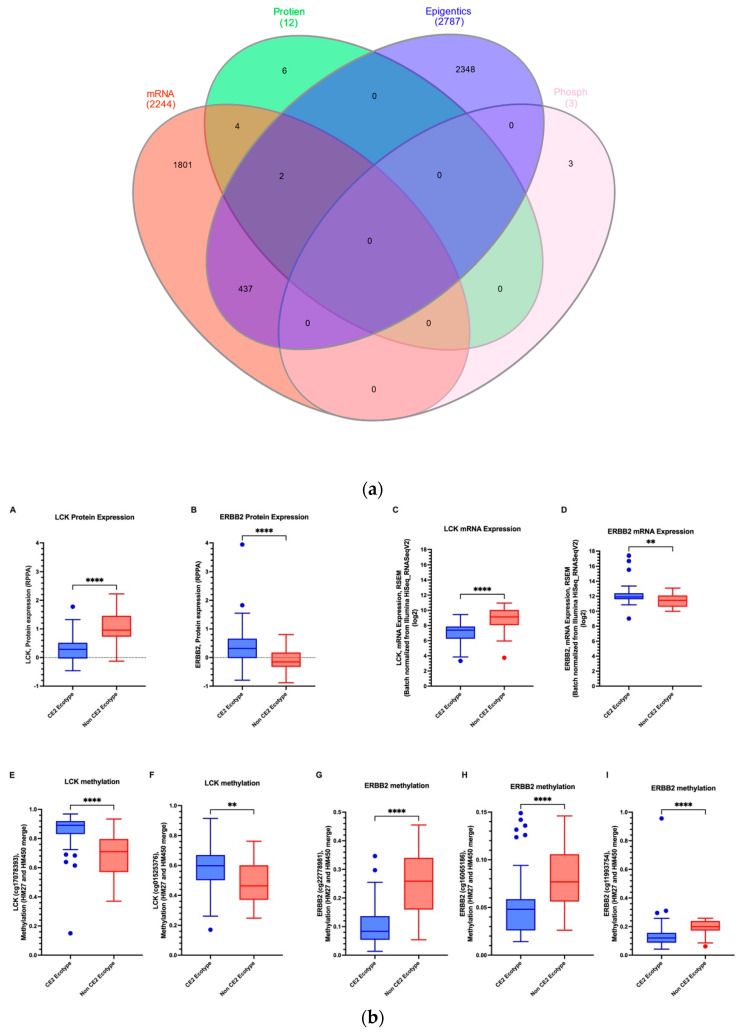
(**a**) mRNA, protein, epigenetic, and methylation expression showed a high number of differential expressions between genes, (**b**) and the mRNA, protein, and methylation expression level of consistently statistically different genes between CE2 and non-CE2 TNBC patients using cBioPortal and TCGA datasets. Asterisk shows statistical significance as follows: ** means *p* ≤ 0.01; **** means *p* ≤ 0.0001; and ns means not significant.

cBioPortal analysis of TNBC CE2 and non-CE2 patients yielded different gene, protein, and epigenetic signatures. Two thousand, two hundred and forty-four genes were differentially expressed, and 2787 genes were differentially methylated between both ecotypes (Figure 7a). On the other hand, 12 proteins significantly differed between the two ecotypes.

Interestingly, only two genes showed consistent changes between the two groups in mRNA, methylation, and protein level (LCK and ERBB2), and four genes showed altered gene and protein levels between the two (PREX1, CASP7, SYK, and ATM).

**Figure 8:**
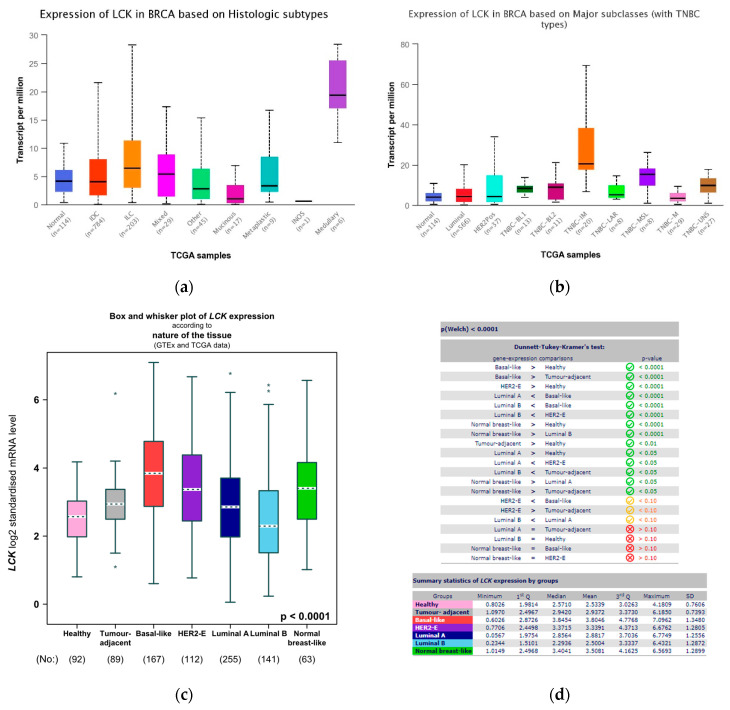
Expression of LCK genes using UALCAN database for BRCA TCGA samples using (**a**) histological subtypes, and (**b**) major subclasses. (**c**) mRNA expression of LCK using both GTEx and TCGA databases. (**d**) Statistical expression of LCK in different breast cancer groups. Asterisk shows statistical significance as follows: * means *p* ≤ 0.05; ** means *p* ≤ 0.01.

LCK plays a key role in promoting TNBC cell growth, survival, and invasion [28], and its expression was found to be upregulated in medullary, TNBC-IM, and basal subtypes (Figure 8a–d).

### 2.8. Correlation between LCK and Neutrophil Activation Markers

Understanding the link between LCK and neutrophils and their correlation is essential. Neutrophils are classified as N1 and N2 and have different states and stages of activation, with each stage exhibiting a unique set of markers.

Using the online bc-GenExMiner, we assessed LCKs’ expression with the neutrophil activation gene to decipher a correlation and link between those genes. The neutrophil activation genes included ITGAM, CZCL8, CXCR2, MMP9, and CEACAM8, as detailed in Table 2. Of these genes, ITGAM was the most significantly correlated with LCK in all subtypes of breast cancer, with the highest correlation occurring in TNBC. 

Furthermore, using the online tool TIMER, we assessed the correlation of LCK with various immune cell infiltrates. The results showed an overall negative correlation between LCK and macrophages, while a positive correlation existed between neutrophils and LCK. CD8+ T cells did not appear to correlate significantly with LCK, as shown in Table 3. This increase in neutrophil correlation could be attributed to the increased correlation between LCK and ITGAM, one of the neutrophil activation markers. 

### 2.9. Neutrophils and Bacteria

Data obtained from BIC database showed the top 10 bacterial compositions in the breast cancer TCGA dataset. Each sample had its immune fractions and bacterial abundance correlated. As bacteria are a source of pathogens and inflammatory stimulation, it is essential to know the responses to each pathogen. Different bacterial species elicit different reactions from neutrophils, with Pseudomonas having the highest correlation with increased neutrophils and Bacteroides decreasing the population of neutrophils, as shown in Table 4. 

Interestingly, looking at the bacterial-associated biological function of Pseudomonas, the pathways involved are linked to inflammation and regulation, as shown in Table 5. However, Bacteroides are involved in metabolic pathways such as ATP synthesis and oxidative phosphorylation. This is in line with the Bacteroides species’ ability to induce specific oncogenic pathways. Though not in the top biological functions of Pseudomonas, six processes are linked to the gene LCK. These processes include leukocyte migration and lymphocyte differentiation. 

### 2.10. Gene Set Analysis

**Figure 9:**
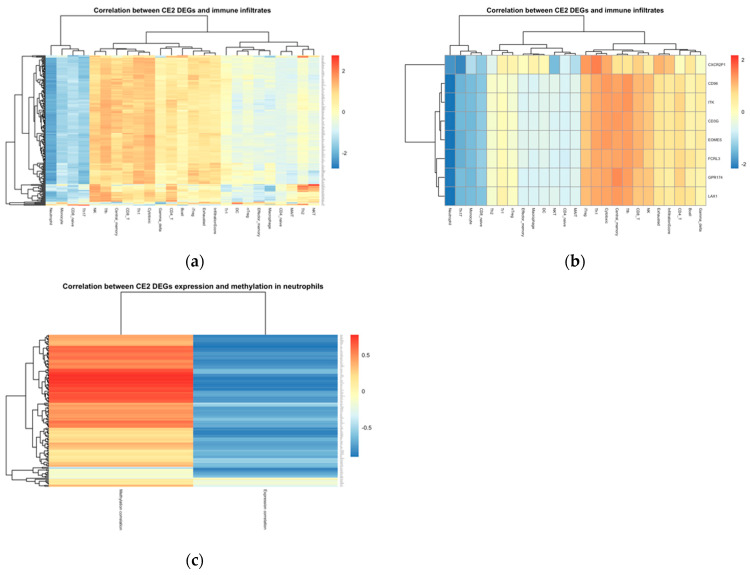
The correlation between the 192 DEGs and their expression in immune infiltrates using GSCA. (**a**) Correlation in all immune infiltrates, (**b**) correlation in only genes that are significantly expressed in all cell types. (**c**) The correlation in gene expression and methylation in neutrophils.

Using the Gene Set Cancer Analysis (GSCA) database, we included the differential expressed genes between CE2 and non-CE2 ecotypes to identify the expression analysis of these genes in response to immune infiltrations. Our results showed that these genes negatively correlated with neutrophils, monocytes, and CD8+ naive cells (Figure 9). From the 197 DEGs, only eight genes had a significant expression level in all cell types in this analysis. Looking into neutrophils’ expression correlation and methylation, we found that only 192 genes were significantly expressed, and 154 were significantly methylated, with 152 genes common between the two. The correlation of these 152 neutrophil-specific genes shows that most genes have a negative correlation in their expression due to the increased methylation they go through.

These 152 genes were then intersected with genes found in normal tissue obtained from the Human Protein Atlas (HPA). Only 15 genes were identified in breast cancer tissues and the neutrophil-specific phenotype. Interestingly, there were two genes that have a difference in their expression between normal breast tissue and breast cancer tissue. GFRA3 (GDNF family receptor alpha 3) and KRT17 (keratin 17) had a different expression in normal and cancer tissues, with GFRA3 expression increasing in breast cancer and KRT17 expression decreasing in breast cancer, as shown in Table 6.

**Figure 10:**
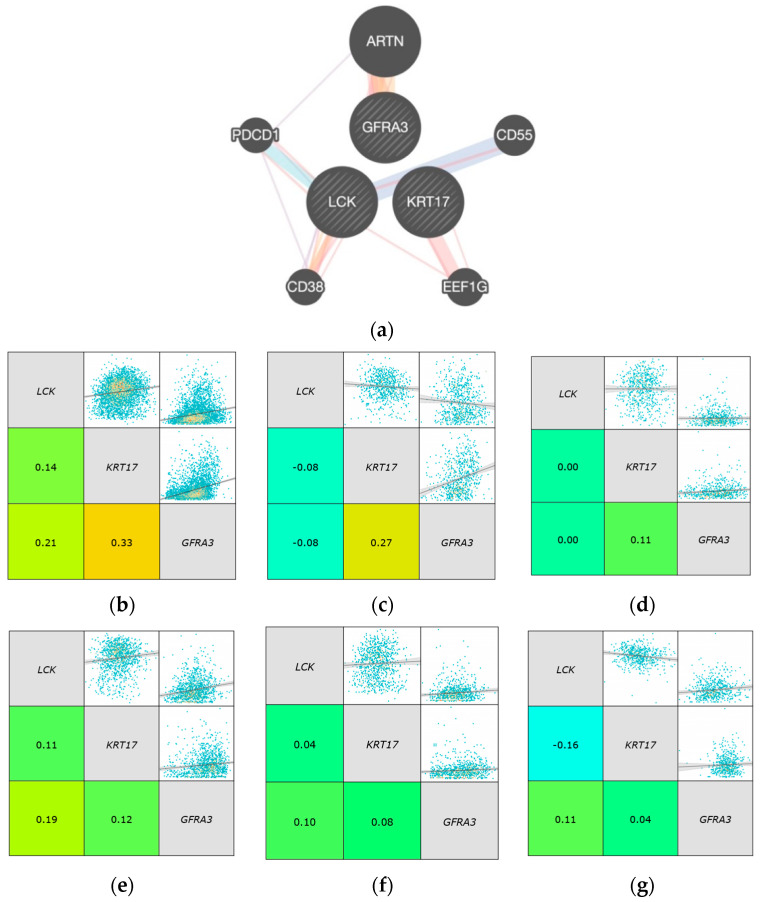
The interaction and correlation between LCK, GFRA3, and KRT17 was identified based on different databases. (**a**) Gene interaction link based on genemania. The correlation of gene expression using bc-GenExMiner in (**b**) all patients, (**c**), TNBC, (**d**) HER2-enriched, (**e**) luminal A, (**f**) luminal B, and (**g**) normal-like breasts.

Using the online tool genemania, we identified a link between LCK and these two genes. LCK physically interacts with the gene EEF1G, which then interacts with KRT17 (Figure 10). In contrast, with GFRA3, LCK interacts with two genes, a physical and co-expression, alongside a pathway interaction with PDCD1, which is co-expressed with ARTN that is in turn co-expressed and has a physical interaction with GFRA3. The other gene it interacts with is CD38, in terms of physical interaction, co-expression, and predicted interactions; CD38 is then co-expressed with PDCD1.

Using bc-GenExMiner, looking at the expression of the three genes in all patients and the PAM50 subtypes, we identified different correlation patterns between the samples. KRT17 and GFRA3 were negatively correlated with LCK in triple-negative breast cancer, while they were positively correlated in other classifications, with the exception of KRT17, which was negatively correlated with LCK in normal-like tissues with the highest correlation level. GFRA3 had the most correlation in the luminal A subtype, as shown in Table 7. 

### 2.11. LCK Gene Expression and the Neutrophil Fraction Are Low in Primary and Metastatic Tissues BC

**Figure 11:**
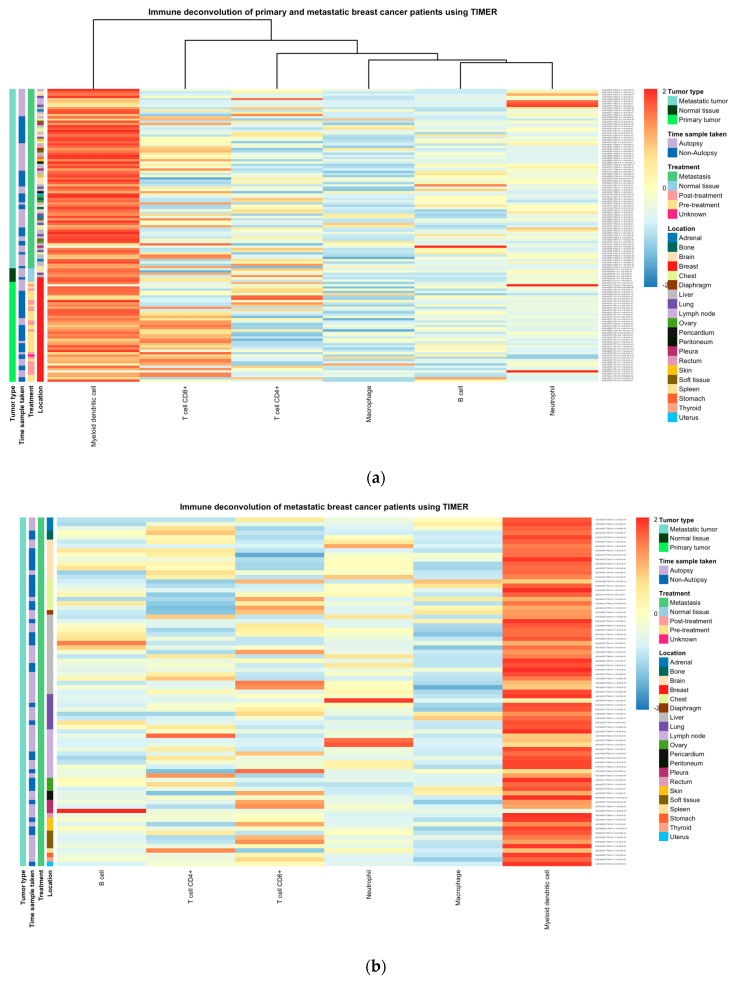

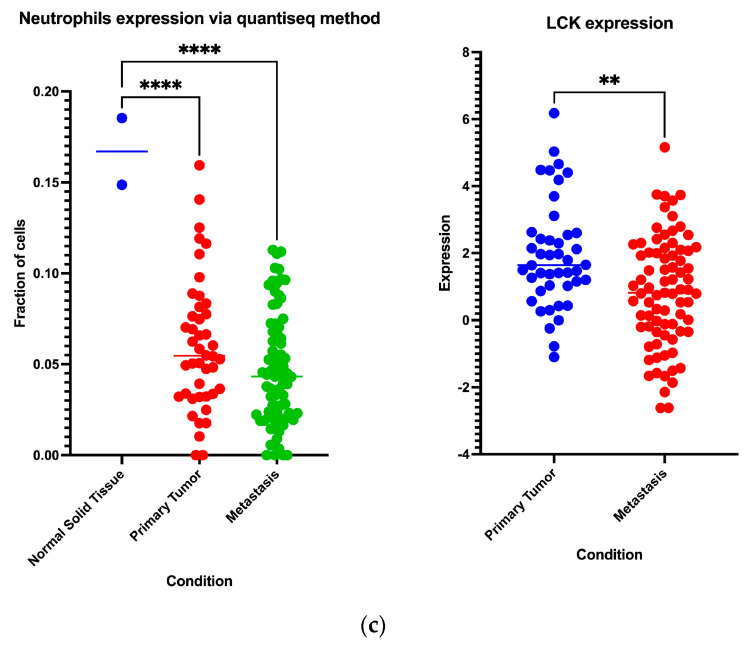
The immune deconvolution and differential gene expression of BC patients showed (**a**) samples clustered with high neutrophils and low monocytes, and (**b**) immune deconvolution of metastatic patients only. (**c**) The relationship between primary tumors showed that metastatic tumors had lower neutrophil fractions and lower LCK expression. Asterisk shows statistical significance as follows: ** means *p* ≤ 0.01; **** means *p* ≤ 0.0001; and ns means not significant.

Immune deconvolution was carried out on RNAseq data obtained from gene expression omnibus (GEO). The dataset GSE209998 sequences RNA from normal tissue, primary tumors, and metastasis. Both primary and metastatic BC showed significantly lower neutrophils than normal sold tissue. Interestingly, metastatic BC showed lower neutrophil infiltration and significantly lower LCK expression, which further confirms the correlation between LCK, neutrophil infiltration, and cancer metastasis and prognosis (Figure 11).

## 3. Discussion

Breast cancer is a complex and heterogeneous disease that affects more than 2 million females worldwide. While the disease affects older women, there has been an increase in incidence rates among younger women in recent years. Various factors play a role in the disease, including its vast immune microenvironment. The role of neutrophils in breast cancer shows that the N1 pro-inflammatory subtype can suppress and attack the tumor. In contrast, the N2 pro tumor subtype aids the tumor in its survival, progression, and metastasis. The tumor microenvironment is rich with different cell types that either lead to a poor or good progression and survival rate. Recently, studies have shown that tumors can be classified based on the ecotype of their microenvironment, with those leukocyte-rich ecotypes having the worst survival [20].

Our reanalysis of publicly available data reveals that while most TCGA patients fall within the CE1 ecotype, the patient stratification differs between subtypes, with most TNBCs being classified within the CE2 subtype. These patients are leukocyte-poor and have four major cell types in their ecotypes: dendritic cells, macrophages, fibroblasts, and epithelial cells. Studies have shown that cancer-associated fibroblasts produce collagen linked to metastasis and tumor progression [29]. Furthermore, the presence of epithelial cells and fibroblasts can be related to the epithelial–mesenchymal transition (EMT), also associated with invasiveness and tumor progression [30].

Further immune deconvolution of these patients revealed that a few cells significantly differ between groups, including macrophages, neutrophils, and T cells. Polymorphonuclear neutrophils (PMNs) originate from the bone marrow during hematopoiesis in response to various cytokines [13,31,32]. Some cytokines are released in response to different stimulants, such as inflammation. Our reanalysis shows that neutrophils appear to be significantly less in normal solid tissue of patients than their tumor cells. Reanalysis of immune cell deconvolution of TNBC patients based on CE2 and non-CE2 subtypes revealed that neutrophil populations were significantly different, with their number being higher in the CE2 ecotype. Additionally, the CE2 ecotype had a high fraction of uncharacterized cells, which included fibroblasts and epithelial cells, among many other cell types.

It is interesting that analysis of gene signatures between the two ecotypes in TNBC patients show that some of the genes highly expressed in the CE2 ecotype included CXCL8, a gene primarily secreted by macrophages. Immune deconvolution had shown that the CE2 ecotype is rich with macrophages, specifically the anti-inflammatory M1 subtype. Other upregulated inflammatory genes in CE2 include IL11, IL17RE, and IL36RN. The genes that are expressed at high levels in CE2 ecotype appear to play a role in various pathways involving cellular organization and development. On the other hand, genes that are significantly higher in non-CE2 ecotypes are involved in immune cell responses, including many interleukins and C-X-C chemokine motifs. This increase in immune-related genes in the non-CE2 ecotype results in different fractions of immune cells circulating in patients and attributing to the types and signatures of microbiomes found in these patients.

All BC showed lower infiltrating neutrophils compared to healthy surrounding tissue. Treated TNBCs improve the count of infiltrating neutrophils in TNBC. Most TNBC patients have a unique CE2 ecotype, characterized by more basal-like epithelial cells, more neutrophils, and fewer mononuclear lymphocytes (B cells, macrophages M1, T cell CD4+ (non-regulatory), and T cell CD8+ and T regs).

The high TIN might block TIL from infiltrating the tumor after being polarized to N2 or low-density neutrophils, which produce arginase one and express the immune checkpoint molecule PD-L1 to inhibit the effector function of T cells [16]. Our analysis showed that CE2 TNBCs cancer cells’ expression of unique genes and proteins was associated with this unique immune cells’ infiltration. Based on that, we tried to show that crosstalk in vitro using cell lines representing TNBC compared to other BC cell lines.

Furthermore, there was an increased correlation between Pseudomonas and neutrophils, which can be part of the associated functions of Pseudomonas. Bacteroides, which have a negative correlation with neutrophils, have been found to induce several oncogenic pathways and are linked to the promotion of breast cancer in mammary ducts. A study has shown that MCF7 cells infected with rhamnolipids produced by Pseudomonas aeruginosa underwent apoptotic cell death [33]. Furthermore, this study has shown that products released by this bacterium can induce cell death of neutrophils, macrophages, and other immune cells [33]. Studies have demonstrated epithelial lymphatic cells secrete chemokines and other factors that increase neutrophil migration towards *p*. aeruginosa [34].

Other studies have shown that the species Bacteroides fragilis can activate oncogenic pathways, including the Wnt/β-catenin pathway [35]. Furthermore, this bacterium is able to induce breast cancer by the colonization of mammary ducts and the promotion of epithelial hyperplasia [35]. This bacterium is able use the various oncogenic pathways to promote tumor proliferation and metastasis [35]. A study has shown that Bacteroides fragilis can induce polarization of macrophages towards the inflammatory M1 phenotype as part of its activation of the innate immune system [36]. Interestingly, several significant pathways were found in Pseudomonas bacteria-associated function involving the gene LCK.

The LCK gene showed significant downregulation in CE2 compared to non-CE2 TNBCs regarding its mRNA and protein, which matched its lower methylation score. Interestingly, the LCK metagene separates the ER-negative group into better or worse prognosis, as there is a strong positive prognostic value for LCK in ER-negative, which seems to respond better to neoadjuvant chemotherapy [37]. On the other hand, the ERBB2 gene showed higher mRNA and protein levels with lower methylation in CE2 TNBCs. It was recently documented that certain TNBCs were not truly HER2 negative [38], and circular RNA (circRNA) ERBB2 is mainly distributed in the cytoplasm of TNBC cells and promotes the proliferation and invasion of TNBC cells [39]. About 34−39% of primary TNBCs show a low expression of ERBB2 [40], and its signaling pathway is activated in the TNBC subtype, but the mechanism is different from the HER+ subtype [41]. Such ERBB2-low status was associated with slightly improved overall survival [42] and had a lower density of TILs compared with ERBB2-negative cancer [43].

Intracellular, non-receptor tyrosine kinase, LCK regulates genes implicated in DNA repair machinery and its attenuated inhibition expression of homologous recombination DNA damage repair genes. Inhibition of LCK has been shown to reduce TNBC cell proliferation and induce cell death, suggesting that LCK could be a potential therapeutic target for TNBC treatment and a potential diagnostic marker [28].

Our results showed that LCK expression was associated with the neutrophil activation genes ITGAM, CZCL8, CXCR2, MMP9, and CEACAM8. Components of the neutrophil activation markers include the surface marker CD11b (ITGAM), which is involved in neutrophil activation, adhesion, and migration [44,45]. Varying levels of CD11b have been observed in both low- and high-density neutrophils, with high levels of CD11b observed in TANs [44]. Some studies have shown that the gene LCK can directly phosphorylate ITGAM at specific tyrosine residues, leading to changes in its conformation and activation of downstream signaling pathways. Phosphorylation of ITGAM by LCK has been shown to enhance neutrophil adhesion and migration by promoting the binding of ITGAM to its ligands, such as intercellular adhesion molecule-1 (ICAM-1) [46]. LCK can also regulate the expression of ITGAM by modulating the activity of transcription factors that control ITGAM gene expression. For example, studies have shown that LCK can activate the transcription factor NF-κB, which plays a critical role in the upregulation of ITGAM expression in response to inflammatory stimuli.

The glycoprotein CD66b (CEACAM8) also exhibits similar properties to CD11b in terms of adhesion and migration towards inflammation [44,45]. CXCR2, a chemokine receptor, is expressed on activated neutrophils to enable migration towards sites of inflammation or infection [47]. IL8 (CXCL8) is a proinflammatory cytokine involved in neutrophil chemotaxis towards inflammation sites and appears to induce NETosis and act on CXCR2 [48]. Matrix metalloproteinase-9 (MMP-9) is involved in NETosis, tissue remodeling, and degradation. It also plays a role in neutrophil migration and infiltration into tissues, with studies showing that MMP9 stimulated tumor angiogenesis [49,50].

On the other hand, neutrophils exhibit polarization markers that differentiate N1 from N2 neutrophils. For example, N2 polarization markers include CD163 and CD206, which are involved in the phagocytosis of apoptotic cells and are also macrophage M2 markers [51]. Another N2 polarization marker is IL-10, an anti-inflammatory cytokine; TGF-β, another cytokine, is involved in tissue repair and fibrosis [52]. Other studies have shown that TGF-β is involved in fibrosis through net formation [53,54]. Arginase-1 is an enzyme produced by N2 neutrophils and can crosstalk to other immune system components, for example, inducing inhibition of T cells by its expression [55].

However, there are common markers between the different subtypes. One of them is myeloperoxidase (MPO), an enzyme involved in generating reactive oxygen species (ROS) and destroying pathogens, with increased CD11b expression being linked to increased MPO release [44]. S100A8 and S100A9 are genes that encode for two calcium-binding proteins involved in host defense mechanisms and inflammation [56,57]. Defensins (DEFA1 and DEFA4) encode for antimicrobial peptides expressed in neutrophil granules and destroy pathogens [58]. Neutrophil elastase (ELANE) is an enzyme involved in the degradation of ECM proteins and the destruction of pathogens, though interestingly, this enzyme has potential antitumor properties [59].

Of these genes, ITGAM is the most heavily correlated with LCK in all subtypes of breast cancer, with the highest correlation occurring in TNBC. Some studies have shown that the gene LCK can directly phosphorylate ITGAM at specific tyrosine residues, leading to changes in its conformation and activation of downstream signaling pathways. Phosphorylation of ITGAM by LCK has been shown to enhance neutrophil adhesion and migration by promoting the binding of ITGAM to its ligands, such as intercellular adhesion molecule-1 (ICAM-1) [46]. Additionally, LCK can also regulate the expression of ITGAM by modulating the activity of transcription factors that control ITGAM gene expression. For example, studies have shown that LCK can activate the transcription factor NF-κB, which plays a critical role in the upregulation of ITGAM expression in response to inflammatory stimuli.

## 4. Methods and Materials

### 4.1. Cancer Databases and In Silico Analysis of Immune Infiltrates

Bulk RNA-seq data of breast cancer were downloaded from The Cancer Genome Atlas (TCGA) database using the TCGA-biolinks package using R software v4.2.2. The online tool Celligner (https://depmap.org/portal/celligner/) and the database cBioPortal (https://www.cbioportal.org/) were used to identify patient BC subtypes and their clinical data. Patient samples were grouped based on subtype and whether treatment was received (Table 8).

The TCGA database yielded 1097 bulk RNA-seq samples for breast cancer. Of these samples, 189 were TNCB samples divided across three groups: treated TNBC, non-treated TNBC, and solid normal tissue from TNBC patients. Eighty patient samples were luminal A, 59 samples were luminal B, and 15 were HER2-enriched breast cancer. Of the 46 normal solid tissue samples, 17 were TNBC, while 29 were from non-TNBC patients, as shown in Table 8. 

Bulk RNA seq data were imported into the online tool EcoTyper (https://ecotyper.stanford.edu/) to identify BC and immune cell ecotypes. TNBC patients were also analyzed based on their ecotype clustering. First, the non-parametric Mann–Whitney test was carried out for ecotype-based immune cell fractions, and *p* ≤ 0.05 was used to indicate statistical significance. Then, samples were analyzed using the immunedeconv package in R Studio and statistical analysis was carried out in GraphPad Prism software V9.5.1. 

### 4.2. Immune Deconvolution of Bulk RNA-Seq Data

**Figure 12:**
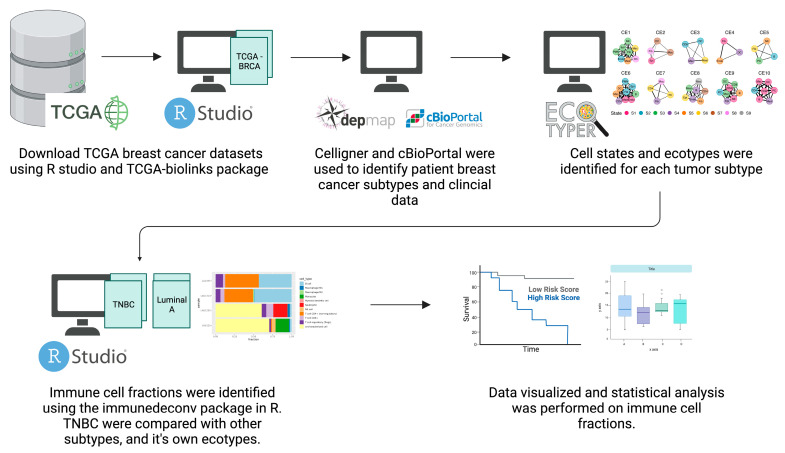
Flow chart of methods used for in silico analysis of TCGA BRCA patients to identify immune cell infiltrates and fractions.

Immune deconvolute uses bulk RNA-seq data against a library of cell signatures to identify immune cell fractions. Immune deconvolution was carried out on datasets obtained from TCGA as well as NCBI’s Gene Expression Omnibus (GEO). Immune deconvolution were carried out using the quantiseq and TIMER methods (Figure 12).

### 4.3. Microbiome Analysis

The Bacteria in Cancer (BIC) database (http://bic.jhlab.tw/) was used to identify the most abundant type of bacterial genus in the breast cancer dataset. The online tool Bacterial Composition was selected, and input included a selection of breast invasive carcinoma as a cancer type, followed by the selection of the taxonomy level and the top number of taxonomies. The relative abundance of each species for each sample was used to correlate the bacterial species with the immune fractions of each patient as obtained from the immune deconvolution. Furthermore, the bacterial-associated biological function was identified for select bacteria from the top 10 relative species.

### 4.4. Differential Expressed Genes and Correlation with Breast Cancer

Further gene level analysis was conducted on grouped TCGA patients using mRNA, protein, epigenetic, and phosphorylation data obtained from cBioPortal. The differential expression of these was selected based on q value ≤ 0.05 and was intersected to identify genes that were differentially expressed across all levels. The differentially expressed genes (DEGs) underwent pathway enrichment analysis using metascape database (https://metascape.org/) to identify enriched pathways, tissue, and cell specificity, as well as transcription factor-target regulatory interactions [60].

The webtool TIMER (http://timer.cistrome.org/) was used to identify the correlation between the common differential expressed on all levels [61]. The gene expression and correlation with various immune infiltrates were analyzed.

The differential expressed genes were used to correlate gene expression with immune infiltrates using the online tool Gene Set Cancer Analysis (GSCA) (http://bioinfo.life.hust.edu.cn/GSCA/#/) [62]. The differentially expressed genes were those selected previously with the addition of a log fold change of ±2. BRCA was selected as the disease of choice, and immune cell abundance was used for single gene level analysis on mRNA expression and methylation on immune infiltrates.

These identified differential expressed genes were then analyzed using the human protein atlas (https://www.proteinatlas.org/) [63,64,65]. Data obtained included expression of a protein in normal tissue and pathology data. The DEGs were used to identify genes and their expression in normal breast tissues. DEGs in normal breast tissue were then compared and their expression was compared in breast cancer tissues. 

The correlation between DEGs whose expression was different in healthy normal tissues and breast cancer was carried out on bc-GenExMiner v4.9 database (http://bcgenex.ico.unicancer.fr/BC-GEM/GEM-Accueil.php?js=1) from the Integrated Oncology Centers [66]. A targeted correlation was performed on the genes using RNA-seq data on all populations and intrinsic molecular subtype (PAM50) populations. Further analyses of interactions of these select genes were carried out using the genemania database (https://genemania.org/) for gene-level analysis and String (https://string-db.org/) for protein-level analysis [67,68].

## 5. Conclusions

We have applied systems biology methods to better understand the immune infiltrates, specifically neutrophils, in breast cancer. Our analysis showed that most TNBC patients in the TCGA-BRCA database were in the CE2 carcinoma ecotype. Neutrophils were more abundant in solid non-tumor tissues in BC patients compared to their tumor tissue and were present at different fractions between tumor subtypes. These differences in cellular fractions between subtypes and ecotypes illustrate the heterogeneity of the disease. LCK has been identified as a gene that plays the role of a master regulator in neutrophil enrichment and polarization towards either pro- or anti-inflammatory states in triple-negative breast cancer.

## Figures and Tables

**Table 1:** TCGA breast cancer patients classified based on their subtype and ecotype.

Carcinoma Ecotype	TNBC (Basal)	% of TNBC	Luminal A	% of Luminal A	Luminal B	% of Luminal B	HER2-enriched	% of HER2-enrcihed	Total
**CE1**	4	4%	115	31%	34	24%	15	29%	168
**CE2**	75	68%	1	0%	3	2%	8	16%	87
**CE3**	2	2%	4	1%	3	2%	0	0%	9
**CE4**	0	0%	1	0%	0	0%	0	0%	1
**CE5**	1	1%	3	1%	1	1%	0	0%	5
**CE6**	0	0%	120	32%	3	2%	0	0%	123
**CE7**	0	0%	3	1%	8	6%	2	4%	13
**CE8**	0	0%	91	24%	51	37%	5	10%	147
**CE9**	24	22%	10	3%	30	22%	14	27%	78
**CE10**	5	5%	26	7%	6	2%	7	14%	44
**Total**	111	100%	374	100%	139	100%	51	100%	675

**Table 2:** Correlation between ITGAM, CXCL8, CXCR2, MMP9, and CEACAM8.

Normal Like	Correlation	Parameters	ITGAM	CXCL8	CXCR2	MMP9	CEACAM8
	LCK	Pearson’s correlation coefficient	0.38	0.03	0.16	0.37	−0.02
	LCK	*p*-value	<0.0001	0.42	0.0001	<0.0001	0.63
	LCK	No. patients	602	602	602	602	602
Lum B	LCK	Pearson’s correlation coefficient	0.42	0.01	0.26	0.29	−0.01
	LCK	*p*-value	<0.0001	0.85	<0.0001	<0.0001	0.75
	LCK	No. patients	966	966	966	966	966
Lum A	LCK	Pearson’s correlation coefficient	0.39	0.06	0.24	0.26	0.02
	LCK	*p*-value	<0.0001	0.0342	<0.0001	<0.0001	0.41
	LCK	No. patients	1343	1343	1343	1343	1343
Her2	LCK	Pearson’s correlation coefficient	0.39	−0.11	0.32	0.19	−0.02
	LCK	*p*-value	<0.0001	0.0032	<0.0001	<0.0001	0.58
	LCK	No. patients	693	693	693	693	693
Basal	LCK	Pearson’s correlation coefficient	0.47	−0.2	0.27	0.19	−0.06
	LCK	*p*-value	<0.0001	<0.0001	<0.0001	<0.0001	0.07
	LCK	No. patients	783	783	783	783	783

**Table 3:** Correlation of LCK expression with different immune infiltrates using TIMER method.

Correlation Table	Parameters	B Cell	Macrophage	Myeloid Dendritic Cell	Neutrophil	T Cell CD4+	T Cell CD8+
BRCA (*n* = 1100)	Spearman’s rank correlation coefficient	0.20	−0.20	0.55	0.54	0.46	0.058
	adjusted *p*-value	<0.0001	<0.0001	<0.0001	<0.0001	<0.0001	0.09
Lum B (*n* = 219)	Spearman’s rank correlation coefficient	0.09	−0.15	0.53	0.53	0.32	0.06
	adjusted *p*-value	0.25	<0.05	<0.0001	<0.0001	<0.0001	0.44
Lum A (*n* = 568)	Spearman’s rank correlation coefficient	0.21	−0.11	0.56	0.49	0.51	0.18
	adjusted *p*-value	<0.0001	<0.05	<0.0001	<0.0001	<0.0001	<0.0001
Her2 (*n* = 82)	Spearman’s rank correlation coefficient	0.24	−0.32	0.54	0.45	0.53	0.23
	adjusted *p*-value	0.05	<0.05	<0.0001	<0.001	<0.0001	0.07
Basal (*n* = 191)	Spearman’s rank correlation coefficient	0.41	−0.22	0.28	0.43	0.36	0.06
	adjusted *p*-value	<0.0001	<0.01	<0.001	<0.0001	<0.0001	0.43

**Table 4:** Bacterial species and their correlation with neutrophil expression.

Bacteria Species	Neutrophil
Pseudomonas	0.27704702
Corynebacterium	0.23749063
Paenibacillus	0.13477395
Acinetobacter	0.10320747
Thermovirga	−0.0176075
Ensifer	−0.0303885
Brevibacillus	−0.0371438
Mycobacterium	−0.0796368
Bacteroides	−0.1681948

**Table 5:** Top 10 bacteria-associated biological functions of Pseudomonas and Bacteroides in breast cancer.

Bacteria	Term	*p* Value	FDR
Pseudomonas	Negative regulation of smooth muscle cell proliferation	0.001	0.037
Pseudomonas	Complement activation	0.001	0.037
Pseudomonas	Negative regulation of blood vessel endothelial cell migration	0.002	0.037
Pseudomonas	Negative regulation of vasculature development	0.001	0.037
Pseudomonas	Ureter development	0.002	0.037
Pseudomonas	Regulation of vasculature development	0.001	0.037
Pseudomonas	C21 steroid hormone metabolic process	0.002	0.037
Pseudomonas	Humoral immune response	0.001	0.037
Pseudomonas	Regulation of positive chemotaxis	0.002	0.037
Pseudomonas	Positive regulation of cholesterol efflux	0.002	0.037
Bacteroides	Cotranslational protein targeting to membrane	0.001	0.136
Bacteroides	ATP synthesis coupled electron transport	0.001	0.136
Bacteroides	NADH dehydrogenase complex assembly	0.001	0.136
Bacteroides	Respiratory electron transport chain	0.001	0.136
Bacteroides	Oxidative phosphorylation	0.001	0.136
Bacteroides	Establishment of protein localization to endoplasmic reticulum	0.001	0.136
Bacteroides	Mitochondrial respiratory chain complex assembly	0.001	0.136
Bacteroides	Mitochondrial electron transport NADH to ubiquinone	0.001	0.136
Bacteroides	Nuclear transcribed mRNA catabolic process nonsense mediated decay	0.001	0.136
Bacteroides	Protein localization to endoplasmic reticulum	0.001	0.136

**Table 6:** Expression of the 15 neutrophil specific genes in breast tissue and in breast cancer cells.

Expression in Normal Breast Tissue				Expression in Breast Cancer				
Gene	Adipocytes	Glandular Cells	Myoepithelial Cells	High	Medium	Low	Not Detected	Present
**PTGDS**	Medium	Medium	Medium	0%	70%	30%	0%	100%
**KLRB1**	Medium	Medium	Medium	0%	75%	25%	0%	100%
**GFRA3**	Low	Low	Low	0%	100%	0%	0%	100%
**GBP4**		Medium	Medium	17%	58%	25%	0%	100%
**ZNF683**		Medium	Low	0%	64%	36%	0%	100%
**SCML4**		Low		0%	0%	91%	9%	91%
**AOAH**		Medium	Medium	10%	70%	10%	10%	90%
**EOMES**		Low	Low	0%	9%	73%	18%	82%
**WDFY4**		Medium	Medium	0%	58%	17%	25%	75%
**CXCR5**		Medium	Low	0%	27%	36%	36%	64%
**PTPN7**		Low		0%	10%	40%	50%	50%
**STAP1**		Low	Low	0%	10%	30%	60%	40%
**KRT17**			High	0%	8%	0%	92%	8%
**SOX10**		Low		0%	0%	0%	100%	0%
**CCR2**		Low		0%	0%	0%	100%	0%

**Table 7:** Correlation between LCK and KRT17 and GFRA3 in all breast cancer samples and in subtypes classified based on PAM50 using bc-GenExMiner.

Correlation with LCK	Parameters	KRT17	GFRA3
All Samples	Pearson’s correlation coefficient	0.14	0.21
	*p*-value	<0.0001	<0.0001
	No. patients	4421	4421
Basal	Pearson’s correlation coefficient	−0.08	−0.08
	*p*-value	0.0352	0.0285
	No. patients	783	783
HER2 enriched	Pearson’s correlation coefficient	0	0
	*p*-value	0.9934	0.9156
	No. patients	693	693
Luminal A	Pearson’s correlation coefficient	0.11	0.19
	*p*-value	0.0001	<0.0001
	No. patients	1343	1343
Luminal B	Pearson’s correlation coefficient	0.04	0.1
	*p*-value	0.1816	0.0017
	No. patients	966	966
Normal Like	Pearson’s correlation coefficient	−0.16	0.11
	*p*-value	0.0001	0.005
	No. patients	602	602

**Table 8:** TCGA breast cancer patient’s groups based on subtype and treatment.

Subtype	Treatment Received	Number of Samples
**TNBC**	Yes	153
**TNBC**	No	19
**Luminal A**	Yes	40
**Luminal A**	No	40
**Luminal B**	Yes	40
**Luminal B**	No	19
**HER2-enriched**	Yes	11
**HER2-enriched**	No	4
**Solid Normal tissue**	N/A	TNBC = 17 Non TNBC = 29

## Data Availability

All generated data is found in the main text of the paper.
